# Editorial and Miscellaneous

**Published:** 1874-08

**Authors:** 


### Editorial and Miscellaneous.

### A CARD FROM THE PUBLISHERS.

The RECORD has, from causes which it would take some time to explain, not made its appearance at the time we contracted with the editors to publish it. In the future, the RECORD will be printed and delivered between the 20th and 25th of each month.

SOUTHERN PUBLISHING COMPANY.

☞ Our thanks are specially due those of our subscribers who have remitted their dues, and thus aided us in carrying out our contract with our publishers. Those who have not done us this favor, have put us to very great inconvenience as well as cost, insomuch as we are their surities for each number sent them. Will they not send their subscriptions promptly, and thereby relieve us during the "hard times?"

☞ Write your Names, Post Office, County and State *plainly*. Be sure to say what Post Office you wish the RECORD sent, and always date your letters. We receive many well written letters, but doubt even the authors being able to decipher their signatures.

☞ Address all Communications, to POWELL & GOLDSMITH, none other.

☞ Send Money by Check, Postal Order or by Registered Letters. We are willing to endorse for the honesty of our subscribers, but cannot be responsible for the irregularity of the mails.

☞ We take pride in the manner in which the SOUTHERN MEDICAL RECORD is put up for mailing. Each number is promptly transmitted to our subscribers by mail, securely wrapped, and plainly addressed.

Our patrons will know therefore, that in case any number should be missing it is not our fault. To accommodate our friends we will endeavor to duplicate any number of the RECORD which may accidentally be *lost* in transmission. P.

### EXTRACTS FROM DR. J. D. TUCKER'S LETTER.

"Though slow in reporting for the RECORD, I have not been idle in working for its promotion. I hope to be able to report a few cases very soon. The RECORD is highly appreciated by the physicians of this part of Tennessee. It is said by all who read it, to be the best and most convenient medical periodical ever published in the United States. Each number furnishes more practical knowledge than six of some of greater pretensions, and a volume of twelve numbers really give the cream of nearly all that is published in this country. This is the way I represent it, and have succeeded in securing over thirty subscribers. If every associate and subscriber would send five, we could not ouly boast of having the best, but the largest circulation of any journal ever published in the United States. Suppose you offer a small premium to all your present subscribers, who will renew their present subscription at the end of this volume and send you two new subscribers."

We sincerely thank our good and hard-working friend for the interest he has taken to aid us in extending the circulation of the RECORD. Similar expressions of approbation came to us from every section of the country; and while our circulation is larger than that of any Medical Journal in the South, proper, this has been the result more of exertion on the part of the editors than of our friends, its readers. We believe our friends when, on every side, they warmly commend it to us by letters, but we—while appreciating their expressions of kindness and appreciation—would be better pleased if they would, individually, go to work to secure a few subscribers each. They can do this. They can, at least, try. We beg them to imitate our friend, Dr. Tucker, and secure at once our list of subscribers.

We expect to place before our readers a list of premiums for the next year, to encourage all to work and subscribe for the RECORD.

THE *Archives of Ophthalmology and Otology*, edited by Professors Knapp and Mood, will hereafter appear, as nearly as possible, every three months. The American edition, under the editorial management of Professor Knapp, will receive also the services of Drs. E. Greunning, of New York, and C. J. Blake, of Boston, the former in the ophthalmological, the latter in the otological department. It will contain, besides original matter, reviews of the current ophthalmological and otological literature.

This periodical is one of the best of its class published in the world, and the only one in the English language, and is deserving of the support of the practitioners in this country, both general and special. It is published by Wm. Wood & Co., New York.

DR. KÖSTER, Professor of Pathological Anatomy, in Geissen, has accepted the scenic chair in Bonn, vice Professor Rendfliesh, who goes to Würzburg.

M. CRUVEILHIER, Professor in the Faculty of Medicine, in Paris, and author of a well-known work on Anatomy, died early in March; aged, eighty-three.

PROFESSOR DU BOIS REYMOND, the eminent Physiolgist, has lately received an invitation to go to Geneva. He has decided not to accept, but will remain at Berlin.

DR. FORBES WINSLOW, who was for many years our highest authority on the brain and nervous system, died in London, on the 4th of March.

IN two weeks twenty sums of 1,000 pounds each were anonymously presented to the various charities of London.

CORRECTION.—Printers, perhaps, are not aware of the great importance of a —, but in optics it amounts to a great deal. If the reader of my article on Astigmotism, in the February number, will place this minus mark before some fractions, on page 75, and make them read thus:—$\frac{1}{10}$ s ⌒ —$\frac{1}{00}$ c and $\frac{1}{30}$ c ⌐ —$\frac{1}{15}$ c, I would not be so liable to a just criticism.

S. M. BURNETT.

